# Tuberculosis transmission in the Indigenous peoples of the Canadian prairies

**DOI:** 10.1371/journal.pone.0188189

**Published:** 2017-11-14

**Authors:** Smit Patel, Catherine Paulsen, Courtney Heffernan, Duncan Saunders, Meenu Sharma, Malcolm King, Vernon Hoeppner, Pamela Orr, Dennis Kunimoto, Dick Menzies, Sara Christianson, Joyce Wolfe, Jody Boffa, Kathleen McMullin, Carmen Lopez-Hille, Ambikaipakan Senthilselvan, Richard Long

**Affiliations:** 1 Department of Medicine, Faculty of Medicine and Dentistry, University of Alberta, Edmonton, Alberta, Canada; 2 School of Public Health, University of Alberta, Edmonton, Alberta, Canada; 3 National Reference Centre for Mycobacteriology, National Microbiology Laboratory, Winnipeg, Manitoba, Canada; 4 Department of Medical Microbiology, University of Manitoba, Winnipeg, Manitoba, Canada; 5 Institute of Aboriginal Peoples’ Health, Canadian Institutes of Health Research, Sudbury, Ontario, Canada; 6 Department of Medicine, College of Medicine, University of Saskatchewan, Saskatoon, Saskatchewan, Canada; 7 Department of Medicine, Max Rady College of Medicine, University of Manitoba, Winnipeg, Manitoba, Canada; 8 Department of Medicine, McGill University, Montreal, Quebec, Canada; 9 Enteric Diseases, National Microbiology Laboratory, Winnipeg, Manitoba, Canada; 10 Division of Bacterial Diseases, National Microbiology Laboratory, Winnipeg, Manitoba, Canada; Agencia de Salut Publica de Barcelona, SPAIN

## Abstract

**Setting:**

The prairie provinces of Canada.

**Objective:**

To characterize tuberculosis (TB) transmission among the Indigenous and non-Indigenous Canadian-born peoples of the prairie provinces of Canada.

**Design:**

A prospective epidemiologic study of consecutively diagnosed adult (age ≥ 14 years) Canadian-born culture-positive pulmonary TB cases on the prairies, hereafter termed “potential transmitters,” and the transmission events generated by them. “Transmission events” included new positive tuberculin skin tests (TSTs), TST conversions, and secondary cases among contacts.

**Results:**

In the years 2007 and 2008, 222 potential transmitters were diagnosed on the prairies. Of these, the vast majority (198; 89.2%) were Indigenous peoples who resided in either an Indigenous community (135; 68.2%) or a major metropolitan area (44; 22.2%). Over the 4.5-year period between July 1^st^, 2006 and December 31^st^ 2010, 1085 transmission events occurred in connection with these potential transmitters. Most of these transmission events were attributable to potential transmitters who identified as Indigenous (94.5%). With a few notable exceptions most transmitters and their infected contacts resided in the same community type. In multivariate models positive smear status and a higher number of close contacts were associated with increased transmission; adjusted odds ratios (ORs) and 95% confidence intervals (CIs), 4.30 [1.88, 9.84] and 2.88 [1.31, 6.34], respectively. Among infected contacts, being Indigenous was associated with disease progression; OR and 95% CI, 3.59 [1.27, 10.14] and 6.89 [2.04, 23.25] depending upon Indigenous group, while being an infected casual contact was less likely than being a close contact to be associated with disease progression, 0.66 [0.44, 1.00].

**Conclusion:**

In the prairie provinces of Canada and among Canadian-born persons, Indigenous peoples account for the vast majority of cases with the potential to transmit as well as the vast majority of infected contacts. Active case finding and preventative therapy measures need to focus on high-incidence Indigenous communities.

## Introduction

Tuberculosis (TB) is an ongoing global public health problem. Within high-income, low-incidence, countries it most commonly affects minority groups and the impoverished. In Canada, for as long as national reports have been filed, Indigenous peoples have had higher rates of TB than any other population group [1]. Moreover, the rate of TB in Indigenous peoples has changed little in recent years despite the progressive decline to near elimination in Canadian-born non-Indigenous people [2]. Given the historical connection between TB in Indigenous peoples in Canada, colonization and the social dimensions of the disease in all population groups, these statistics are troubling [3]. In 1982, the *Constitution Act of Canada* recognized three major groups of Indigenous peoples: First Nations, who may be registered or un-registered with the federal government under the terms of the *Indian Act*, Métis, self-identified persons of mixed Indigenous and non-Indigenous ancestry, and Inuit [4]. According to the Public Health Agency of Canada (PHAC), TB cases among the First Nations and Métis population in the prairie provinces of Alberta, Saskatchewan and Manitoba contribute more than one-half of the total Indigenous TB cases in all of Canada [2].

With respect to the foreign-born in Canada, TB elimination strategies emphasize the prevention of active TB in those with latent TB infection (LTBI), as most transmission is understood to have taken place overseas. With respect to Canada’s Indigenous peoples, TB elimination strategies emphasize both the interruption of transmission and the prevention of active TB in those with LTBI, as virtually all transmission is understood to have taken place in country. Among Indigenous peoples TB tends to be localized to selected high incidence communities and the inner city where it is believed that the reservoir of LTBI is constantly being replenished by ongoing transmission [5–7]. Herein we report the findings of a prospective study of TB transmission in the Canadian-born Indigenous and non-Indigenous populations of the prairie provinces. Consecutive adults with the potential to transmit were identified in 2007–2008 and their transmission events (newly infected contacts with or without disease) described over the 4.5 years from July 1, 2006 to December 30, 2010. The findings are part of a mixed-methods study of TB on the Canadian prairies: *“the Determinants of TB transmission in the Canadian-born population of the prairie provinces*,” or “*DTT project*” [8]. By documenting where, by whom, and to whom transmission is occurring we seek to inform national TB elimination strategies.

## Methods

### Ethical considerations and relationships

The *DTT project* was a patient-based study that followed an extensive engagement and ethical approval process with multiple jurisdictions including both Indigenous and non-Indigenous stakeholders. In anticipation of a large number of persons recruited into this study identifying as Indigenous peoples, the study followed the Canadian Institutes of Health Research (CIHR) Guidelines for Health Research Involving Aboriginal peoples. These guidelines were the primary, and mandatory minimum for conducting any health research projects with Indigenous peoples when this study began. Over and above these minimum requirements, the project was overseen by Provincial Network Committees (PNCs), one in each participating province, that were established for the purpose of conducting the study and interpreting the results. The PNCs were comprised of former patients, Elders, traditionalists, community-based tuberculosis workers, and health researchers. The process of establishing these committees and acquiring the administrative and ethical approvals is described in detail elsewhere [8]. Briefly, site principal investigators, Indigenous scholars, and other stakeholders in each province organized their own PNCs. They also obtained their own home university and program ethics and administrative approvals. PNCs met individually and together at intervals throughout the life of the project. Their travel and honoraria were supported by the budget of the operating grant. Institutional level ethics approvals were received from Health Canada and the Prairie Universities of Alberta, Calgary, Saskatchewan and Manitoba.

### Study setting and community type

**Fig 1:**
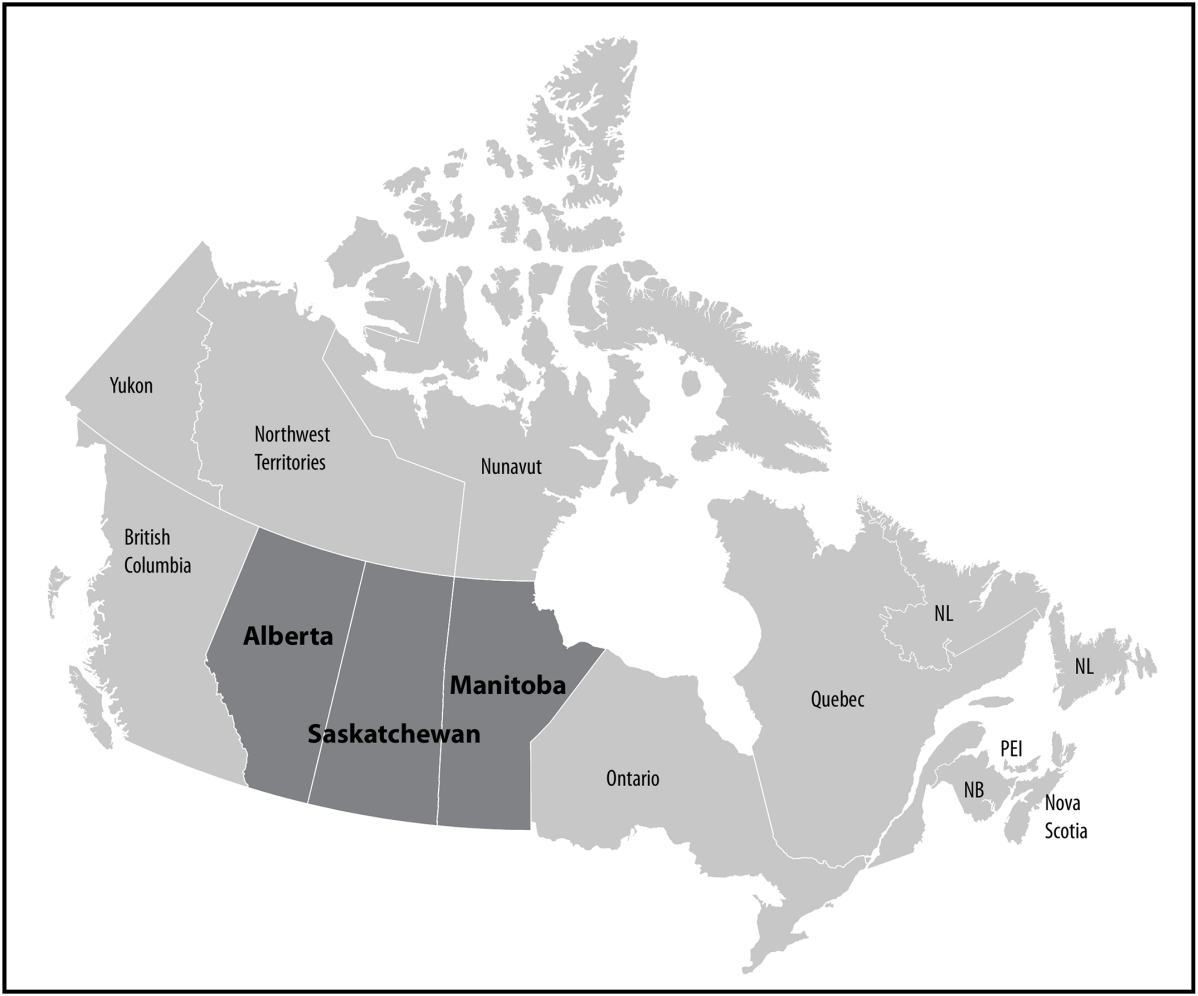
Map of Canada highlighting the three prairie provinces, Alberta, Saskatchewan and Manitoba. For perspective, the country of France is about one-third the size of the three prairie provinces combined (0.643M km^2^) and in 2006 had a population that was 12x as large (63.62M)–see http://data.worldbank.org/indicator/SP.POP.TOTL?location=FR. Abbreviations: NL Newfoundland; NB New Brunswick; PEI Prince Edward Island.

The study setting included the three prairie provinces of Canada which together cover a large geographic area (1.781M km^2^) and have a relatively small population (5.34M in 2006, Statistics Canada), see Fig 1. In our analysis communities-of-residence of cases and contacts were defined as: i) major metropolitan area (the major cities of Winnipeg [Manitoba], Regina and Saskatoon [Saskatchewan] and Edmonton and Calgary [Alberta], ii) non-major metropolitan area (smaller communities with ≥500 persons that were neither Métis settlements/communities nor reserve communities), iii) Métis settlement/community, or iv) reserve community. Métis settlements/communities and reserve communities in each province have previously been defined independent of this study [7].

### Potential TB transmitters and their contacts

This study began with the identification and description of consecutive “potential TB transmitters” on the prairies in 2007–2008, defined as Canadian-born persons over the age of 14 years diagnosed with culture-positive (i.e. their sputum was culture-positive for *Mycobacterium tuberculosis*) pulmonary TB. Interested members of this cohort then met with a provincial study coordinator who elicited written informed consent to participate in a quantitative survey. Potential transmitters whose sputum was positive for acid-fast bacilli on smear and therefore likely to be more infectious, were also invited to participate in a qualitative interview. Contacts of potential transmitters included all close household or non-household and casual contacts, as defined by the Canadian TB Standards [9]. Contact tracing was performed as part of routine service provision using the concentric circle approach whereby those contacts closest to the source case are screened first and additional contacts screened in ever widening circles depending upon the extent of transmission seen in the last circle [10]. Transmission events included any new positive tuberculin skin tests (TSTs) and TST conversions as defined by the Standards and all secondary cases. Secondary cases refer to persons diagnosed with primary disease in the six months prior to the date of diagnosis of the potential transmitter with whom they had been in contact (i.e. the secondary case, not the potential transmitter, was the index case) or with primary or post-primary disease within the twenty-four months following the date of diagnosis of the potential transmitter with whom they had been in contact. In this study the date of diagnosis was defined as the start date of treatment or date of death in the event the diagnosis was made after death. Secondary cases were categorized as “*Type 1”* if they were culture positive and had both conventional and molecular epidemiologic links and “*Type 2”* if they were culture-negative and had only conventional epidemiologic links to the potential transmitter presumed to be the source of their infection [11, 12]. In the event that a contact was shared by more than one potential transmitter, transmission attribution was based upon the most plausible scenario—which was in turn based upon transmitter (the presence or absence of cough, smear status, cavitation status) and contact (close, casual) characteristics and the timing of the event relative to the date of diagnosis of the potential transmitter.

To ensure that all *Type 1* secondary cases in each potential transmitters 30-month transmission window were identified and to address other study-related questions, initial isolates from all culture-positive TB cases on the prairies over the 4.5-year period, July 1, 2006 to December 31, 2010, were DNA fingerprinted. Genotyping was performed using 24-loci Mycobacterial Interspersed Repetitive Units (MIRU)-Variable Number Tandem Repeat (VNTR) by the National Reference Centre for Mycobacteriology, National Microbiology Laboratory in Winnipeg, Manitoba [13, 14]. Isolates had to have matched on all available loci. A 30-month transmission window was chosen because the risk of disease after infection is highest during this period of time [15, 16]. For purposes of the analysis, and to estimate the reproductive number, i.e. the number of secondary pulmonary cases generated per potential transmitter, those “potential transmitters” that met the original inclusion criteria but who were subsequently determined to be secondary cases of another potential transmitter, were removed from the final list of potential transmitters.

### Data sources and data elements

Data was collected from respective provincial TB Registries and, among those who consented, a quantitative survey. Data on potential transmitters included age, sex, population group, community-of-residence, sputum smear status, chest radiograph appearance (cavitary or non-cavitary), education level, employment status, and persons-per-room in the home they reported living in at the time of diagnosis. Data on contacts included age, sex, population group and community-of-residence.

### Data analysis

Demographic characteristics of potential transmitters and infected contacts are described according to community-of-residence. The distribution of infected contacts is described according to the population group and community-of-residence of their potential transmitter.

Potential transmitters were grouped according to the transmission events they generated as follows: (i) those who had one or more versus no transmission events and (ii) those who had high versus low “transmission scores.” Transmission scores relate to the certainty with which the transmission event could be attributed to the potential transmitter as follows: new positive TST = 1; TST conversion = 2; *Type 2* secondary case = 3; *Type 1* secondary case = 4. Transmission outcomes were analyzed by comparing those with and without transmission events, and those with high and low transmission scores.

The “attack rate,” or proportion of infected contacts that were secondary cases, was calculated according to age, sex, population group, community-of-residence and contact type (close versus casual/other).

### Statistical methods

Multiple logistics regression was used to determine the significance of the association between dichotomous outcome (number of transmission events ≥1 vs none; LTBI vs active disease) and other factors taken together [17]. These factors include demographic and geographic characteristics, population group and clinical characteristics. We used the purposeful selection method for model building. First, a univariate logistic regression model was fitted for each independent variable. An interim multivariate model was fitted including all of the independent variables that were significant in the univariate logistic regression models at p = 0.20, and which had less than 80% missing data. A higher p-value of 0.20 was chosen to allow for the confounding effects of variables that are not statistically significant at p≤0.05. Clinically significant variables were included and retained, regardless of statistical significance.

The cavitation variable was missing data for approximately 18% of the potential transmitters. We repeated the logistic regression analysis for transmission risk with the same variables, cavitation excepted, that were used in the primary analysis. All statistical analyses were conducted using STATA 12.0.

## Results

**Fig 2:**
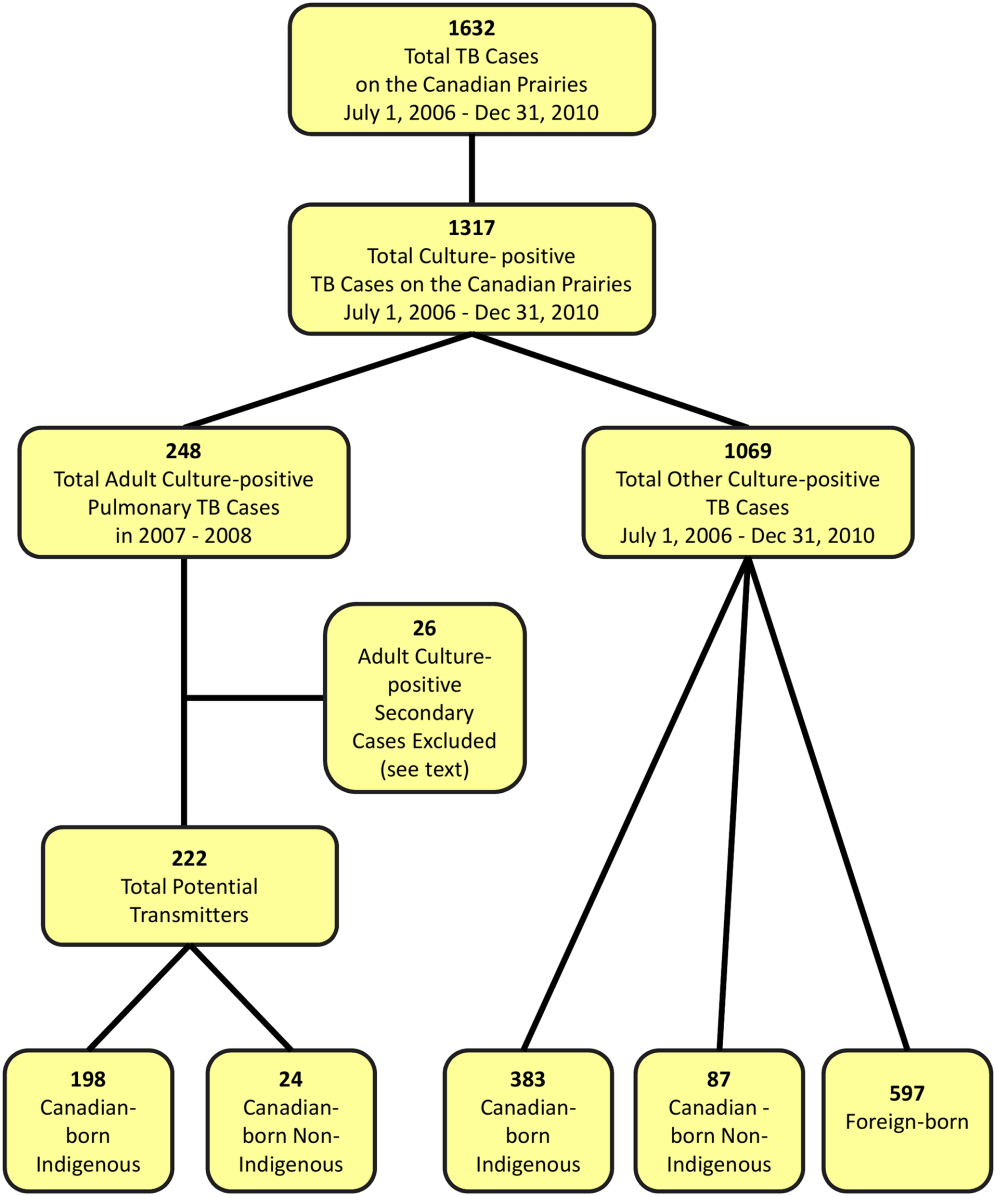
Tuberculosis cases in the prairie provinces of Canada between July 1, 2006 and December 31, 2010. Population group information was missing for two of the 1069 “other” culture-positive cases; of the 26 cases meeting the original study inclusion criteria and designated a secondary case, 25 were Indigenous and 1 was non-Indigenous.

Over the 4.5-year period, July 1, 2006 to December 31, 2010, 1632 patients, 1317 culture-positive, were diagnosed with TB on the prairies (Fig 2). Of the 1317 culture-positive cases 248 were Canadian-born, ≥14 years of age and diagnosed in 2007–2008 with pulmonary TB. Of these 248 adult Canadian-born culture-positive pulmonary cases, 26, 25 Indigenous and 1 non-Indigenous, were found to be secondary cases of another potential transmitter in the total group of 248. Compared to the other 222 adult Canadian-born culture-positive pulmonary cases these 26 secondary cases were significantly less likely to be coughing (p<0.03), be smear-positive (p<0.001) or have cavitation on chest radiograph (p<0.001), see online supplement. They are hereafter considered secondary cases; the other 222 hereafter remain potential transmitters. The age, sex and population group of these potential transmitters is described in Table 1 according to their community-of-residence. Most potential transmitters were young or middle-aged (91.0%), Registered First Nations or Métis (89.2%) and residing in either an Indigenous community (61.3%) or a major metropolitan area (27.0%). Distribution by age and population group differed across community type. Canadian-born non-Indigenous cases were few in number (24 or 10.8%) and were most likely to live in major metropolitan areas (67.0%). Altogether there were more male than female potential transmitters (58.6% vs 41.4%) and distribution across community types was similar.

**Table 1:** Demographic characteristics of potential transmitters by community-of-residence*.

Characteristic	All communities n	Community-of-residence			
		Reserve Communities n (%)	Métis communities n (%)	Major metropolitan n (%)	Non-major metropolitan n (%)
**No. Assessed**	222	112	24	60	26
**Age (years)**					
15 to 34	82	51 (62)	11 (13)	12 (15)	8 (10)
35 to 64	120	54 (45)	10 (8)	43 (36)	13 (11)
≥65	20	7 (35)	3 (15)	5 (25)	5 (25)
**Sex**					
Male	130	64 (49)	14 (11)	39 (30)	13 (10)
Female	92	48 (52)	10 (11)	21 (23)	13 (14)
**Population Group**					
Registered First Nations	158	108 (68)	1 (1)	34 (22)	15 (9)
Métis/Other Indigenous	40	4 (10)	22 (55)	10 (25)	4 (10)
Canadian Non-Indigenous	24	0 (0)	1 (4)	16 (67)	7 (29)

* See text for definition of population groups and communities-of-residence.

The age, sex, and population group of infected contacts is described in Table 2 according to community-of-residence. Most infected contacts were children or young and middle-aged adults (95.9%), just as likely to be male as female (52.5% vs 47.5%), and among those whose population group was known, likely to be either Registered First Nations or Métis (87.3%). Very few foreign-born persons were infected by Canadian-born potential transmitters (35 or 3.5%). Distribution of the infected contacts differed across communities by age, sex and population group. The majority of infected Registered First Nations or Métis contacts were living rurally in an Indigenous community (77.1%) or a non-major metropolitan area (17.7%).

**Table 2:** Demographic characteristics of infected contacts by community-of-residence*.

Characteristic^†^	All communities n	Community-of-residence			
		Reserve Communities n (%)	Métis communities n (%)	Major metropolitan n (%)	Non-major metropolitan n (%)
**No. Assessed**	**1076**^‡^	**593**	**96**	**158**	**229**
**Age (years)**					
<15	301	195 (65)	39 (13)	20 (7)	47 (16)
15 to 34	352	198 (56)	35 (10)	44 (13)	75 (21)
35 to 64	378	182 (48)	22 (6)	82 (22)	92 (24)
>64	44	17 (39)	0 (0)	12 (27)	15 (34)
Unknown	1	1 (100)	0 (0)	0 (0)	0 (0)
**Sex**					
Male	556	334 (60)	44 (8)	72 (13)	106 (19)
Female	504	246 (49)	52 (10)	83 (16)	123 (24)
Unknown	16	13 (81)	0 (0)	3 (19)	0 (0)
**Population Group**					
Registered First Nations	817	572 (70)	58 (7)	43 (5)	144 (18)
Métis/Other Indigenous	59	7 (12)	38 (64)	3 (5)	11 (19)
Canadian Non-Indigenous	93	2 (2)	0 (0)	46 (49)	45 (48)
Foreign-Born	35	1 (3)	0 (0)	22 (63)	12 (34)
Unknown	72	11 (15)	0 (0)	44 (61)	17 (24)

* Infected contacts include all contacts who were identified as being a secondary case or of having either a new positive tuberculin skin test or a tuberculin skin test conversion.

^†^ See text for definition of population groups and communities-of-residence.

^‡^ There were 1085 transmission events in total. Nine of these contacts were lacking information on community-of-residence.

When data were combined for potential transmitters, infected contacts and community-of-residence in Tables 3 and 4, it became evident that most infected contacts, regardless of population group, were infected by Registered First Nations or Métis source cases (94.5%) and that all but a few of the infected Registered First Nations and Métis contacts were infected by Registered First Nations and Métis potential transmitters (99.4%). Further in about two-thirds of the transmission events (63% to 72%), the potential transmitter and infected contact were from the same community type. Place-of-residence of potential transmitters and infected contacts also suggested mobility between reserve communities and both Métis communities and non-major metropolitan areas and between major metropolitan areas and reserve communities.

**Table 3:** Distribution of infected contacts by population group of their potential transmitter.

Population group of infected contacts	Total Assessed	Population Group of Potential Transmitter		
		Registered First Nations n (%)	Métis / Other Indigenous n (%)	Canadian-Born Non-Indigenous n (%)
**Total Assessed**	1085	837	188	60
**Population Group**				
Registered First Nations	823	757 (90)	62 (33)	4 (7)
Métis / Other Indigenous	60	15 (2)	44 (23)	1 (2)
Canadian Non-Indigenous	93	31 (4)	32 (17)	30 (50)
Foreign-Born	35	9 (1)	10 (5)	16 (27)
Unknown	74	25 (3)	40 (21)	9 (15)

**Table 4:** Distribution of infected contacts by community-of-residence of their potential transmitter.

Community-of-residence of infected contacts	Total Assessed	Community-of-Residence of Potential Transmitter			
		Reserve Community n (%)	Métis Community n (%)	Major Metropolitan n (%)	Non-Major Metropolitan n (%)
**Total Assessed**	1085	715	60	182	128
**Community-of-Residence**					
Reserve Community	593	513 (72)	10 (17)	40 (22)	30 (23)
Métis Community	96	47 (7)	42 (70)	0 (0)	7 (5)
Major Metropolitan	158	24 (3)	1 (2)	125 (69)	8 (6)
Non-Major Metropolitan	229	125 (17)	7 (12)	17 (9)	80 (63)
Unknown	9	6 (1)	0 (0)	0 (0)	3 (2)

In Table 5 the potential transmitters with transmission events are compared to those with none for a range of demographic, geographic, clinical and socio-economic characteristics. In univariate logistic regression analysis, age, community-of-residence, smear status, chest radiograph, number of close contacts per case, and number of persons-per-room were all associated with one or more transmission event. In a multivariable model that included age, population group, sex, community-of-residence, smear status, radiograph appearance, and number of close contacts per case, having a positive smear or a larger number of close contacts were associated with one or more transmission event and being female or living in a Métis community were associated with having no transmission events. Living in a non-major metropolitan area, having a positive smear, cavitation on chest radiograph and a larger number of close contacts were associated with a higher transmission score (S2 Table).

**Table 5:** Demographic, geographic and clinical characateristics of potential transmitters by whether or not they generated one or more transmission event.

Characteristic	Total Assessed n (%)	Number of Transmission Events		Logistic Regression	
		≥ 1 n (%)	None n (%)	Univariate Odds Ratio*	Ajusted Odds Ratio*
**No. Assessed**	222	168	54		
**Age (years)**					
15 to 34	82	69 (84)	13 (16)	1.0	1.0
≥35	140	99 (71)	41 (29)	**0.45 [0.23, 0.91]**	0.49 [0.22, 1.10]^†^
**Sex**					
Male	130	102 (78)	28 (22)	1.0	1.0
Female	92	66 (72)	26 (28)	0.70 [0.78, 1.29]	**0.37 [0.17, 0.80]**
**Population Group**					
Canadian Non-Indigenous	24	18 (75)	6 (25)	1.0	1.0
Registered First Nations	158	123 (78)	35 (22)	1.17 [0.43, 3.18]	1.55 [0.41, 5.84]
Métis/Other Indigenous	40	27 (68)	13 (33)	0.69 [0.22, 2.16]	1.85 [0.37, 9.36]
**Community-of-Residence**					
Reserve community	112	90 (80)	22 (20)	1.75 [0.85, 3.61]^‡^	1.66 [0.64, 4.30]
Métis Community	24	13 (54)	11 (46)	0.51 [0.19, 1.34] ^‡^	**0.22 [0.05, 1.00]**
Major metropolitan	60	42 (70)	18 (30)	1.0	1.0
Non-Major metropolitan	26	23 (88)	3 (12)	3.29 [0.87, 12.35]^†^	4.03 [0.91, 17.83]^†^
**Smear Status**					
Negative	84	52 (62)	32 (38)	1.0	1.0
Positive	138	116 (84)	22 (16)	**[3.24, 1.72, 6.12]**	**4.30 [1.88, 9.84]**
**Chest Radiograph**					
Cavitary	82	72 (88)	10 (12)	**3.04 [1.38, 6.68]**	2.35 [0.92, 6.05] ^†^
Non-Cavitary	101	71 (70)	30 (30)	1.0	1.0
Unknown	39	25 (64)	14 (36)	0.75 [0.35, 1.65]	0.50 [0.20, 1.22]
**No. of Close Contacts**^§^					
<9	112	74	38	1.0	1.0
≥9	110	94	16	**3.02 [1.56, 5.83]**	**2.88 [1.31, 6.34]**
**Employed at diagnosis**					
Yes	63	48 (76)	15 (24)	0.89 [0.42, 1.88]	
No	101	79 (78)	22 (22)	1.0	
Unknown	58	41 (71)	17 (29)	(omitted)	
**Education**					
No High School Diploma	140	109 (78)	31 (22)	1.0	
High School Diploma or More	23	18 (74)	6 (26)	0.81 [0.29, 2.22]	
Unknown	59	41 (69)	18 (31)	(omitted)	
**Crowding (PPR)**					
≤1	52	35 (67)	17 (33)	1.0	
>1	71	60 (85)	11 (15)	**2.65 [1.12, 6.29]**	
Not Applicable^¶^	39	31 (79)	8 (21)	(omitted)	
Unknown	60	42 (70)	18 (30)	(omitted)	

* Odds Ratios highlighted in bold are significant at p<0.05.

^†^ Indicates odds ratios significant at p<0.10.

^‡^ Indicates odds ratios significant at p<0.20.

^§^ Number of close contacts was dichotomized using the median number of contacts.

^¶^ These participants did not live in either a house or condominium. Note: Apartments and hotels were grouped together in the survey, thus apartments were not included. Twenty-one participants reported living in an apartment or hotel. Shared accommodations and rooming houses were also excluded (n = 5).

The proportion of infected contacts who were identified as being either *Type 1* or *Type 2* secondary cases is described in Table 6. The reproductive number, i.e. the ratio of the number of new adult culture-positive pulmonary cases generated (n = 70) over the number of potential transmitters (n = 222), was 0.32. The percent probability at which infected contacts became active TB cases (“attack rate”) was 11.6%. Compared to infected Canadian-born non-Indigenous people, infected Registered First Nations and Métis were more likely to progress to disease. Infected contacts in the age group 35–64 years were less likely than those in the age group 0–14 years to progress to disease. Infected contacts who were living in reserve communities or non-major metropolitan areas were less likely to have disease than infected contacts who lived in major metropolitan areas. Infected close contacts were more likely than casual contacts to have disease.

**Table 6:** Proportion of infected contacts, by age, sex, population group, community-of-residence and contact type (close vs casual/other) that were secondary cases (“attack rate”).

Characteristic	All Infected contacts n (%)	Disease Type		Attack Rate^†^ % [95% CI]	P-Value	Adjusted Odds Ratio^‡^
		LTBI n (%)	Active Disease* n (%)			
**Total Assessed**	1085	959 (88.4)	126 (11.6)	--	--	991
**Age Group (years)**						
<15	301	247 (82.1)	54 (17.9)	17.9 [13.6, 22.3]	REF	1.0
15 to 34	355	317 (89.3)	38 (10.7)	10.7 [7.5, 13.9]	**0.008**	0.65 [0.41, 1.04]^§^
35 to 64	384	351 (91.4)	33 (8.6)	8.6 [5.8, 11.4]	**0.0003**	**0.61 [0.37, 1.00]**
>64	44	43 (97.7)	1 (2.3)	2.3 [0, 6.7]	**0.008**	0.16 [0.02, 1.23]^§^
Unknown	1	1 (100.0)	0 (0.0)			
**Sex**						
Male	560	491 (87.7)	69 (12.3)	12.3 [9.6, 15.0]	REF	1.0
Female	508	451 (88.8)	57 (11.2)	11.2 [8.5, 14.0]	0.58	0.84 [0.56, 1.24]
Unknown	17	17 (100.0)	0 (0.0)			
**Population Group**						
Registered First Nations	823	722 (87.7)	101 (12.3)	12.3 [10.0, 14.5]	**0.049**	**3.59 [1.27, 10.14]**
Métis/Other Indigenous	60	41 (68.3)	19 (31.7)	31.7 [19.8, 43.5]	**<0.0001**	**6.89 [2.04, 23.25]**
Canadian Non-Indigenous	93	88 (94.6)	5 (5.4)	5.4 [0.8, 10.0]	REF	1.0
Foreign-born	35	34 (97.1)	1 (2.9)	2.8 [0, 8.5]	0.55	0.50 [0.05, 4.49]
Unknown	74	74 (100.0)	0 (0.0)			
**Community-of-Residence**						
Reserve Communities	593	536 (90.4)	57 (9.6)	9.6 [7.2, 12.0)	0.65	**0.29 [0.14, 0.57]**
Métis Communities	96	68 (70.8)	28 (29.2)	29.2 (20.0, 38.3)	**0.0002**	0.78 [0.34, 1.80]
Major Metropolitan	158	141 (89.2)	17 (10.8)	10.8 (5.9, 15.6)	REF	1.0
Non-Major Metropolitan	229	205 (89.5)	24 (10.5)	10.5 (6.5, 14.5)	0.93	**0.39 [0.18, 0.81]**
Unknown	9	9 (100.0)	0 (0.0)			
**Contact Type**						
Close	567	490 (86.4)	77 (13.6)	13.6 (10.8, 16.4)	REF	1.0
Casual/Other	518	469 (90.5)	49 (9.5)	9.5 (6.9, 12.0)	**0.034**	**0.66 [0.44, 1.00]**
**Smear Status of Source**						
Negative	204	190 (93.1)	14 (6.9)	6.9 (3.4, 10.3)	REF	1.0
Positive	881	769 (87.3)	112 (12.7)	12.7 (10.5, 14.9)	**0.019**	1.79 [0.97, 3.29]^§^

* Active disease cases include those identified by contact tracing investigations. There were 70 *type I* secondary cases, 50 over the age of 14 years and pulmonary, and 56 *type II*.

^†^ The attack rate refers to the proportion of new infections (the total of new positive TSTs, TST conversions and secondary cases) that were secondary cases in the 30-month transmission window of their source.

^‡^ Odds ratios highlighted in bold are significant at p<0.05.

^§^ Indicates odds ratios significant at p<0.10.

REF: Reference group.

## Discussion

Over the two years 2007–2008 there were 222 Canadian-born potential TB transmitters, among whom almost 90% identified as Indigenous (89.2%: 71.2% Registered First Nations, 18.0% Métis), who resided in either an Indigenous community (reserve or Métis community) (68.2%) or a major metropolitan area (22.2%). Cumulatively, over the 30 months that constituted individual transmission windows, these potential transmitters gave rise to 1085 transmission events. Most transmission events were attributed to Indigenous potential transmitters (94.5%). Most transmission appeared to be local (63–72%), i.e. transmitter and infected contact were from the same community type, although some mobility between reserve and both Métis communities and non-major metropolitan communities and between major metropolitan communities and reserves, was suggested. Potential transmitters with one or more transmission event were more likely to be smear-positive or have a larger number of close contacts and less likely to be female or living in a Métis community compared to a major metropolitan area. The proportion of infected contacts that had disease (i.e. the “attack rate”) was higher in both Registered First Nations and Métis than in the Canadian-born non-Indigenous and lower in those age 35–64 years compared to those age 0–14 years, in those living in reserve communities or non-major metropolitan areas compared to those living in major metropolitan areas and in those who were casual/other contacts compared to close contacts.

Truly remarkable about these findings is the extent to which incident pulmonary disease and transmission was limited to Indigenous Canadian-born persons who, during the study period, represented only 11.0% of the Canadian-born population in the prairies (Statistics Canada). Only one Canadian-born non-Indigenous potential transmitter was diagnosed per month and they accounted for only 5.5% of the transmission events. While the results of this particular study speak only to TB in the prairies, the aforementioned disparity of this preventable and curable disease which proliferates among the impoverished is a national phenomenon as evidenced by the PHAC-published “TB in Canada” reports. A sustained high incidence of TB in the Indigenous peoples of Canada, in combination with a falling incidence in the Canadian-born non-Indigenous, i.e. increasing rate ratios, is a story that has been in the telling for many years [18]. If what we call ‘structural violence’ is either that which increases the distance, or impedes the decrease of the distance, between the potential (the burden of disease in the non-Indigenous) and the actual (the burden of disease in the Indigenous), then, with respect to TB and its causes, it may be said that an act of violence has been/is being experienced by Indigenous peoples in Canada [19].

Although the number of secondary pulmonary cases generated per potential transmitter (the reproductive number) was less than one (see Table 6), suggesting that current programmatic activity will eventually lead to the eradication of the disease, that will not happen soon and certainly not at the pace necessary to achieve elimination targets [20, 21]. Instead we call for intensified case finding aimed at those most likely to transmit, and treatment of LTBI in those most likely to progress to disease. In our study, progression to disease may have resulted from delayed or incomplete evaluation of contacts, missed opportunities for initiation or difficulties with completing therapy among the latently infected [22], a more infectious environment [23], or host factors including nutritional deficiencies, co-morbidities and possibly genetic predisposition [9, 24–28]. Our study makes no distinction in this regard. This and earlier studies, suggest that the efficiency of intensified activity may be maximized by focusing on communities that are reporting a sustained high incidence of disease [5–7,29–31]. They indicate that Indigenous communities and major metropolitan areas are heterogeneous with respect to TB incidence; in Indigenous communities a finding that may relate to the local history of post-contact TB epidemics, with those communities experiencing more remote epidemics, i.e. before the 1920’s, having a lower contemporary burden of disease and less transmission [32].

To work within and across high incidence Indigenous and non-Indigenous communities will require the building of respectful relationships. These relationships must bridge jurisdictional lines and truly engage communities as partners, taking the word “partner” beyond a euphemism for a relationship among people with unequal power that it sometimes represents [33]. As the “biological expression of social inequality” TB demands that responsive efforts adopt a biosocial approach and be prepared to advocate [34, 35]. For Indigenous peoples this means addressing not just classic socioeconomic and connectivity deficits, the most striking of which is the ongoing crisis in housing, which appears to have contributed to transmission [36, 37] but also Indigenous-specific determinants related to colonization, globalization, immigration, loss of language and culture and disconnection from the land [38].

Strengths of this study include the completeness of the conventional and molecular epidemiologic data and the methods by which we have attributed transmission events to potential transmitters [11, 12]. Limitations include missing quantitative survey data (some patients did not consent or if they did consent did not complete all questions), missing chest radiograph information for some cases, and population group information for some contacts along with data that would allow us to determine the relative contribution of factors that maintain the disease among Indigenous peoples (e.g. host vs environment vs contact). Mathematical models are known to be beneficial in understanding infectious disease dynamics [39–41]. More specifically, mathematical modeling (dynamic and stochastic) has been used in the analysis of TB transmission dynamics in Indigenous populations [42,43]. The impact of improving follow-up during a contact investigation and preventive therapy for LTBI have been investigated using mathematical modeling [42–45]. In the context of the *DTT Project*, an analysis of TB transmission using agent-based modelling is being applied in a separate communication.

In summary, this and earlier studies strongly suggest that Indigenous peoples are bearing an inequitable burden of TB among the Canadian-born of the Canadian prairies. Data suggest that, with a few notable exceptions related to specific patterns of mobility, disease and transmission is highly focal. Thus, eliminating TB in these communities or regions of high incidence will require more collaborative and intensified activity.

## Supporting information

S1 Table Demographic characteristics of “true” potential transmitters and “false” potential transmitters, i.e. those considered to be more appropriately categorized as secondary cases. (DOCX)

S2 Table Demographic, geographic and clinical characteristics of potential transmitters by transmission score.* (DOCX)
