# Supplementary material for: Tuberculosis transmission in the Indigenous peoples of the Canadian prairies
Source: PLoS One. 2017 Nov 14;12(11):e0188189. doi: 10.1371/journal.pone.0188189 (PMC5685619; doi:10.1371/journal.pone.0188189)
Supplement: S1 Table — (DOCX) [file pone.0188189.s001.docx]

**TABLE S1. DEMOGRAPHIC CHARACTERISTICS OF “TRUE” POTENTIAL TRANSMITTERS AND “FALSE” POTENTIAL TRANSMITTERS, I.E. THOSE CONSIDERED TO BE MORE APPROPRIATELY CATEGORIZED AS SECONDARY CASES**

| Characteristic | Total Assessed  N (%) | “True” Potential Transmitters  N (%) | “False” Potential Transmitters  N (%) | p-value |
| --- | --- | --- | --- | --- |
| **No. Assessed** | 248 | 222 | 26^*^ |  |
| **Smear Status**  Negative  Positive | 103  145 | 84  138 | 19  7 | **0.001** |
| **Chest Radiography**  Non-Cavitary  Cavitary  Unknown | 123  82  43 | 101  82  39 | 22  0  4 | **<0.001** |
| **Syptomatology**  Presence of Cough  Absence of Cough  Unknown | 144  58  46 | 132  47  43 | 12  11  3 | **0.031** |

* Of the 26 pulmonary TB cases that met the original inclusion criteria but were excluded on account of being a secondary case, 7 met all three criteria for non-infectiousness (no cough, smear-negative, non-cavitary), 13 met two criteria, 3 met one criteria, and in 3 the number of criteria was unknown. Eleven of these 26 pulmonary cases, all Indigenous, generated 63 transmission events; 61 in Indigenous contacts, 1 in a Canadian-born non-Indigenous contact and 1 in a contact whose population group was unknown.
