# Supplementary material for: Tuberculosis transmission in the Indigenous peoples of the Canadian prairies
Source: PLoS One. 2017 Nov 14;12(11):e0188189. doi: 10.1371/journal.pone.0188189 (PMC5685619; doi:10.1371/journal.pone.0188189)
Supplement: S2 Table — (DOCX) [file pone.0188189.s002.docx]

**TABLE S2. DEMOGRAPHIC, GEOGRAPHIC AND CLINICAL CHARACATERISTICS OF POTENTIAL TRANSMITTERS BY TRANSMISSION SCORE**^*^

| **Characteristic** | **Total Assessed**  **n (%)** | **Transmission Score** | | **Logistic Regression** | |
| --- | --- | --- | --- | --- | --- |
|  |  | **High Transmitters** | **Low Transmitters** | **Univariate**  **Odds Ratio**^†^ | **Adjusted**  **Odds Ratio**^†^ |
| **No. Assessed** | 222 | 111 | 111 | 222 | 222 |
| **Age (years)**  15 to 34  ≥35 | 82  140 | 48 (59)  63 (45) | 34 (41)  77 (55) | 1.0  0.58 [0.33, 1.01] ^‡^ | 1.0  0.72 [0.38, 1.38] |
| **Sex**  Male  Female | 130  92 | 63 (48)  48 (52) | 67 (52)  44 (48) | 1.0  1.16 [0.68, 1.98] | 1.0  0.77 [0.40, 1.46] |
| **Population Group**  Canadian Non-Indigenous  Registered First Nations  Métis/Other Indigenous | 24  158  40 | 10 (42)  82 (52)  19 (48) | 14 (58)  76 (48)  21 (53) | 1.0  1.51 [0.63, 3.60]  1.27 [0.46, 3.52] | 1.0  1.69 [0.50, 5.69]  1.97 [0.49, 7.96] |
| **Community-of-Residence**  Reserve community  Métis community  Major metropolitan  Non-Major metropolitan | 112  24  60  26 | 61 (54)  9 (38)  21 (35)  20 (77) | 51 (46)  15(63)  39 (65)  6 (23) | **2.22 [1.16, 4.25]**  1.11 [0.42, 2.97]  1.0  **6.19 [2.15, 17.8]** | 1.71 [0.74, 3.94]  0.56 [0.14, 2.16]  1.0  **6.27 [1.89, 20.88]** |
| **Smear Status**  Negative  Positive | 84  138 | 30 (36)  81 (59) | 54 (64)  57 (41) | 1.0  **2.55 [1.46, 4.48]** | 1.0  **2.49 [1.23, 5.07]** |
| **Chest Radiograph**  Cavitary  Non-Cavitary  Unknown | 82  101  39 | 55 (67)  46 (46)  10 (26) | 27 (33)  55 (54)  29 (74) | **2.44 [1.33, 4.46]**  1.0  **0.41 [0.18, 0.93]** | 1.88 [0.92, 3.86] ^‡^  1.0  **0.30 [0.12, 0.74]** |
| **No. of Close Contacts**^§^  <9  ≥9 | 112  110 | 41 (37)  70 (64) | 71 (63)  40 (36) | 1.0  **3.03 [1.75, 5.23]** | 1.0  **2.80 [1.47, 5.34]** |
| **Employed at diagnosis**  Yes  No  Unknown | 63  101  58 | 31 (49)  58 (57)  22 (38) | 32 (51)  43 (43)  36 (62) | 0.72 [0.38, 1.35]  1.0  (omitted) |  |
| **Education**  No High School Diploma  High School Diploma or More  Unknown | 140  23  59 | 76 (54)  12 (52)  23 (39) | 64 (46)  11 (48)  36 (61) | 1.0  0.92 [0.38, 2.22]  (omitted) |  |
| **Crowding (PPR)**  ≤1  >1  Not Applicable^¶^  Unknown | 52  71  39  60 | 28 (54)  41 (58)  19 (49)  23 (38) | 24 (46)  30 (42)  20 (51)  37 (62) | 1.0  1.17 [0.57, 2.41]  (omitted)  (omitted) |  |

* Transmission scores are defined in the text.

† Odds Ratios highlighted in bold are significant p<0.05.

‡ Indicates odds ratios significant p<0.10.

§ Number of close contacts was dichotomized using the median number of contacts.

¶ Participants did not live in a house or condominium. Note: Apartments and hotels were grouped together in the survey, thus apartments were not included. 21 participants reported living in an apartment or hotel. Shared accommodations and rooming houses were also excluded (n=5).
